# Using Breast Cancer Gene Expression Signatures in Clinical Practice: Unsolved Issues, Ongoing Trials and Future Perspectives

**DOI:** 10.3390/cancers13194840

**Published:** 2021-09-28

**Authors:** Romain Varnier, Christophe Sajous, Solène de Talhouet, Colette Smentek, Julien Péron, Benoît You, Thibaut Reverdy, Gilles Freyer

**Affiliations:** 1 Medical Oncology Department, Hôpital Lyon Sud, Institut de Cancérologie des Hospices Civils de Lyon (IC-HCL), Université Claude Bernard Lyon 1, 69310 Lyon, France; christophe.sajous@chu-lyon.fr (C.S.); solene.de-talhouet@chu-lyon.fr (S.d.T.); julien.peron@chu-lyon.fr (J.P.); benoit.you@chu-lyon.fr (B.Y.) ; thibaut.reverdy@chu-lyon.fr (T.R.); gilles.freyer@univ-lyon1.fr (G.F.); 2 Laboratoire Parcours Santé Systémique, EA 4129, Université Claude Bernard Lyon 1, 69372 Lyon, France; colette.smentek@univ-lyon1.fr; 3 Laboratoire de Biométrie et Biologie Evolutive, Equipe Biostatistique-Santé, CNRS UMR 5558, Université Claude Bernard Lyon 1, 69622 Villeurbanne, France; 4 EA3738, CICLY & CITOHL, Université Claude Bernard Lyon 1, 69310 Lyon, France

**Keywords:** breast cancer, gene expression signature, genomic assay, clinical trials

## Abstract

**Simple Summary:**

Gene expression signatures were initially developed to take into account tumor biology for adjuvant chemotherapy decision and have become a standard option in hormone receptors-positive/HER2-negative early breast cancer. While recent randomized phase III studies have provided high level evidence to support their use, much more remains to be explored. This prospective review highlights the unsolved issues regarding targeted populations, delineates the best clinical indications and addresses questions that ongoing and future trials will have to meet. Apart from adjuvant chemotherapy indications, we review their potential interest to tailor neoadjuvant systemic treatments, adjuvant radiation therapy, extended adjuvant hormone therapy and CDK4/6 inhibitor adjuvant treatment.

**Abstract:**

The development of gene expression signatures since the early 2000′s has offered standardized assays to evaluate the prognosis of early breast cancer. Five signatures are currently commercially available and recommended by several international guidelines to individualize adjuvant chemotherapy decisions in hormone receptors-positive/HER2-negative early breast cancer. However, many questions remain unanswered about their predictive ability, reproducibility and external validity in specific populations. They also represent a new hope to tailor (neo)adjuvant systemic treatment, adjuvant radiation therapy, hormone therapy duration and to identify a subset of patients who might benefit from CDK4/6 inhibitor adjuvant treatment. This review will highlight these particular issues, address the remaining questions and discuss the ongoing and future trials.

## 1. Introduction

Despite manifest progresses in the comprehension of breast cancer biology, the decision to administer neoadjuvant or adjuvant systemic treatments remains challenging. The decision making process used to rely on traditional prognostic factors such as lymph node involvement, tumor size, tumor grade and immunohistochemical-based markers for hormone receptors (HR) and HER2 expression [1]. In the era of personalized treatments, these factors appeared insufficient for optimum decision making.

The development of gene expression signatures since the early 2000′s offered standardized assays to evaluate the risk of recurrence for women with HR-positive/HER2-negative (HR+/HER2−) early breast cancer, and therefore helped to individualize the treatment decisions. Five gene expression signatures are currently commercially available and frequently used: OncotypeDX © (Genomic Health Inc., Redwood City, CA, USA), MammaPrint © (Agendia BV, Amsterdam, the Netherlands), Prosigna © (NanoString Technologies, Seattle, WA, USA), EndoPredict © (Myriad Genetics Inc., Salt Lake City, UT, USA) and Breast Cancer Index © (bioTheranostics INC., San Diego, CA, USA) [2]. They have been validated in different retrospective studies such as the ABCSG 6/8, NSABP B14/B20/B28 and TransATAC cohorts and are not interchangeable (main characteristics and validation cohorts are summarized in Table 1). Despite providing broadly equivalent risk information at a populational level, they may provide different risk information for an individual patient [3,4,5]. Several recently published phase III trials (summarized in Table 2) provided high level of evidence data [6,7,8] but many questions still remain unanswered.

The aim of this prospective review is to review data available in the literature about the different assays, as a way of understanding their strengths and drawbacks along with the targeted populations. These data will enable delineating the best clinical indications and discuss the unsolved issues that should be addressed in ongoing and future studies. To reach such an aim, we (1) reviewed the characteristics of the different assays and recall evidence from recent phase III studies, (2) compared their prognostic and predictive abilities, (3) highlighted the need for validation studies in specific populations, (4) discussed their interest to tailor indications for hormone therapy, radiation therapy and CDK4/6 inhibitors in the neo-adjuvant and adjuvant settings, and (5) summarized real-life evidence and cost-effectiveness analyzes. Major ongoing trials are depicted in Table 3.

## 2. Development and Validation of Gene Expression Signatures

Molecular signatures were initially developed in breast cancer to stratify patients according to their prognosis and commonly provided a risk estimation for recurrence and/or death. The goal was to spare chemotherapy to patients with intrinsic excellent prognosis. Besides this prognostic role, they appeared to be useful to predict the differential benefit of adjuvant chemotherapy [1].

Prognostic biomarkers inform about the probability of an outcome (e.g., risk estimation for recurrence and/or death), independent of treatment received [9]. They can be useful to spare chemotherapy to patients with intrinsic excellent prognosis, or to increment adjuvant treatment for patients with poor prognosis. Predictive biomarkers predict response to specific therapeutic intervention [9]. They are critical to identify patients likely to benefit, and to spare the others from unnecessary side effects.

The individualized approach of gene expression signatures must be differentiated from algorithm-based models such as “Adjuvant! Online” and PREDICT [10,11]. The “individual prediction” is based on a population-based algorithm, rather than a genuine personalized approach, and does not take into account the intrinsic genotype of the tumor.

Cancer biomarker development involves multiple steps, including analytical validation (accuracy, reproducibility, standardization) and clinical utility validation (i.e., ability to detect the genotype of interest and to support the best treatment decision) [12]. The five commercially available gene expression signatures were initially validated in “prospective-retrospective” studies (summarized in Table 1) using archived samples (assayed after study completion) and patient data from prospective trials which were not initially designed to evaluate the assay.

A retrospective pooled database analysis showed that patients classified as high-risk with MammaPrint © assay benefited from chemotherapy, contrary to low-risk patients for whom the benefit was not significant [13]. Sestak and colleagues showed that patients with a high EPclin score (a score taking account both EndoPredict © results and clinical parameters) who received chemotherapy had a significantly lower recurrence risk than those who received hormone therapy alone, suggesting that a high EPclin score can predict chemotherapy benefit [14]. Furthermore, the EPclin score was shown to predict absolute chemotherapy treatment benefit in the adjuvant setting [15]. At least two studies showed statistically significant results with the OncotypeDX © assay for both node-negative and node-positive early breast cancers, with a larger benefit from adjuvant chemotherapy for women with higher recurrence scores [16,17].

Translational data demonstrated that each signature was differently determined by hormonal-receptor features or proliferation markers [18], and therefore might be more appropriate to differentially predict hormone therapy or chemotherapy benefit. Future studies will have to readdress the question at a time when hormone therapy tends to be extended over 5 years and when systemic treatments are incremented or decremented according to the tumor subtype and pathological response to neoadjuvant therapy.

## 3. Contribution of Recent Randomized Phase III Trials to Current Knowledge

It is increasingly recognized that “prospective-retrospective” designed studies represent acceptable level of evidence, although dedicated prospective randomized clinical trials remain the gold standard to validate clinical utility [19].

Three randomized phase III trials recently provided level 1 evidence for the MammaPrint © and OncotypeDX © signatures [6,7,20]. These studies varied in their design, eligibility criteria and objectives, which are briefly summarized in Table 2 (see [21] for in-depth comparison and analysis).

### 3.1. Prognostic Ability

All three studies corroborated prognostic ability of the two gene expression signatures. The MINDACT trial achieved it primary objective with a 94.7% (95%CI, 92.5–96.2%) 5-year distant metastasis-free survival for high-clinical risk/low-genomic risk patients untreated with chemotherapy [6]. An exploratory analysis evaluated an additional threshold for patients with “ultralow” risk within the low-risk category: their prognosis appeared excellent with a 8-year distant metastasis-free rate of 95–98% [22]. The TAILORx trial showed that patients with low-genomic risk (recurrence score ≤ 11) had an excellent prognosis treated with HT alone (9-year invasive disease-free survival, 84% ± 1.3%) [7]. In addition, recurrence risk and overall survival were significantly associated with genomic recurrence score in the global population. The WSG-planB trial similarly reported a 94.2% (95%CI, 91.2–97.3%) 5-year invasive disease-free survival for patients with low-genomic risk (recurrence score ≤ 11) treated with HT only [20].

### 3.2. Predictive Ability

In all three trials, the assays played an important role in assigning the treatment, but only the MINDACT and TAILORx studies prospectively evaluated their predictive value regarding to chemotherapy in the randomized arms. The MINDACT study did not demonstrate significant differences for any outcomes between chemotherapy-treated and untreated patients in the discordant groups, and therefore did not support a predictive role for the MammaPrint © signature in this situation [6]. The TAILORx study showed no significant difference in 9-year IDFS between chemotherapy-treated and untreated patients in the intermediate group (recurrence score 11–25) [7]. However, subgroup analyzes suggested a potential benefit for women ≤50 years with recurrence score 16–20 and women with recurrence score 21–25.

### 3.3. Clinical Utility

The MINDACT trial confirmed the prognostic ability of MammaPrint © signature for patients with invasive breast cancer, regardless of their hormone-receptor expression, HER2-status and nodal involvement [6]. Patients with clinical-high risk but genomic-low risk tumors can probably be spared from adjuvant chemotherapy.

In a lymph node-negative HR+/HER2−population, the TAILORx trial suggested that adjuvant chemotherapy could be omitted for patients with recurrence score ≤ 25, with a potential concern for intermediate-high risks (recurrence score 21–25) and patients aged < 50 with recurrence score 16–25 [7]. Finally, the WSG-planB trial, showed that adjuvant chemotherapy could also be omitted for patients with up to three involved lymph nodes when recurrence score is ≤11 [20]. Preliminary results of the RxPONDER trial also supported omission of adjuvant chemotherapy for post-menopausal women with up to three involved lymph nodes and a recurrence score ≤ 25 [23].

## 4. Can We Apply Established Gene Expression Signatures to All Populations?

### 4.1. Premenopausal Women

The most compelling evidence was first provided in postmenopausal women. The TAILORx and RxPONDER trials then evaluated the OncotypeDX © recurrence score also in premenopausal women [7,23]. Exploratory analyses of the TAILORx study suggested some benefit of chemotherapy for women aged under 50 years with a recurrence score of 16 to 25 [7], and this benefit seemed to peak at 45 years of age in premenopausal women [24]. The preliminary reported results of the RxPONDER study also suggested a benefit of chemotherapy for non-menopaused women with a ≤25 recurrence score [23]. However, only 15% of premenopausal patients were treated with ovarian suppression. Rather than a better chemosensitivity in non-menopausal women, the iatrogenic amenorrhea ovarian suppression might indirectly explain this better outcome [25]. It remains unclear whether chemical ovarian suppression could offer similar benefit, as shown in the TEXT and SOFT trials [26].

A recent meta-analysis aimed to evaluate the use of genomic signatures in young (≤40 years) breast cancer patients [27]: young patients classified as low-risk had a higher tendency to receive chemotherapy compared to their older counterparts, while their excellent prognosis would potentially have permitted them to avoid adjuvant chemotherapy. A dedicated prospective study might improve confidence for sparing chemotherapy administration to low-genomic risk young patients.

Current knowledge remains insufficient to rely on gene expression signatures to predict ovarian suppression usefulness nor to compare it benefit with adjuvant chemotherapy. However, chemotherapy might safely be omitted for patients <50 years with an OncotypeDX © recurrence score ≤ 15.

### 4.2. Elderly Patients

Age remains the main risk factor for breast cancer, and about 30% of breast cancers are diagnosed in women older than 70 years [28]. Observational studies suggested a more favorable tumor biology, including higher rates of hormone-receptors expression and lower rates of HER2 overexpression, but larger tumors in elderly patients [29]. In this frailer population, gene expression signatures could help in individualizing treatment decisions and avoid toxic treatments. Patients over 60 years old accounted for about 30% of patients included in the validation cohorts of OncotypeDX © assay [16,17]. The TAILORx trial included 27.7% patients aged from 61 to 70 years but only 4.9% patients aged over 70 [7].

Two retrospective studies specifically evaluated the effect of the 21-gene recurrence score in real life older patients: the distribution of the recurrence score was similar between older and younger patients and its prognostic value was confirmed; however both studies failed to demonstrate any survival improvement with chemotherapy in high-risk elderly patients [30,31].

The GERICO 11/ASTER 70s phase III trial investigated the benefit of tailored adjuvant systemic treatment according to the Genomic Grade test (derived from frozen MapQuantDx™, Ipsogen) in women aged over 70 [32]. High genomic-risk patients were randomized between hormonal therapy alone or chemotherapy followed by hormonal therapy, whereas low genomic-risk or contra-indicated patients were followed in an observational parallel cohort receiving hormone therapy alone. The overall survival (OS) being the primary endpoint, this more stringent criteria will allow us to better understand who among the older patients may benefit from adjuvant chemotherapy and who may not.

There is no concern about using of gene expression signatures for elderly patients inside validated indications.

### 4.3. Non-Caucasian Populations

The main established gene expression signatures were initially developed and validated in patients enrolled in North American and European trials [33,34,35,36]. Theses samples included a large majority of Caucasian people and might not represent the ethnic diversity of populations. Yet, breast cancer is associated with specific molecular factors [37] and a worse prognosis [38,39] in African American women, and the luminal B subtype seems to be overrepresented in Asian women [40]. The question whether the commercialized gene expression signatures can be applicable to non-Caucasian populations remains unclear, with some data suggesting risk overestimation in these populations [41].

Cheng and colleagues developed the first Asian-based gene expression signature, with a 18-gene signature able to predict both locoregional recurrence and distant metastasis [42]. Preliminary results of a head-to-head comparison to OncotypeDX © recurrence score indicated a >80% correlation rate in Chinese patients [43], but a longer follow-up is warranted.

Rather than development of dedicated signatures, the validation of established signatures in specific populations might be a more pragmatic approach.

However, initial evidence indicates that commercially available gene expression signatures might overestimate genomic risk in non-Caucasian patients, and therefore decrease their ability to avoid chemotherapy. On the other hand, their ability to identify high-risk patients seems to be preserved, which minimizes the risk for suboptimal treatment.

## 5. Can We Tailor Hormone Therapy Decisions?

Hormone therapy remains the backbone of systemic adjuvant therapy for hormone receptor-positive (HR+) breast cancer, with a classical duration of 5 years. However, HR+ breast cancers have a proclivity for late recurrence, which can occur after 5 years of adjuvant hormone therapy [44]. The risk of late recurrence is partially correlated to the tumor characteristics and node extension—tumors with the worse prognosis have a peak of relapse between 5 and 8 years—but these criteria are insufficient to predict who might benefit from extended hormonal adjuvant therapy [45].

### 5.1. Prognosis Evaluation beyond 5 Years of Hormone Therapy

In the absence of dedicated predictive factors, some authors have evaluated established gene expression signatures to identify very good prognosis patients who can be spared from extended hormone therapy. The prognostic role of Prosigna ©/PAM50 risk-of-recurrence (ROR) score on predicting late recurrences in HR+ node-positive and node-negative breast cancer was determined by Sestak et al., using long-term follow-up data and tissue samples from the Arimidex, Tamoxifen Alone or in Combination (ATAC) and Austrian Breast and Colorectal Cancer Study Group 8 (ABCSG 8) clinical trials [45,46]. In the same way, the prognostic role of the 21-gene OncotypeDX © recurrence score was established in high-expressing Estrogen Receptor (ESR1) breast cancers based on results from the National Surgical Adjuvant Breast and Bowel Project (NSABP) B-28 and B-14 clinical trials [47] and independently from ESR1 expression in the TransATAC cohort [48].

The EndoPredict © score is a multigene assay specifically developed in patients who only received 5 years of hormone therapy (without adjuvant chemotherapy) in the ABCSG6 and ABCSG8 trials [34]. It integrates expression levels of both proliferative and estrogen receptor signaling genes, which are associated with early (0–5 years) and late (5–10 years) recurrences, respectively, and can be associated with clinical parameters in the EPclin score [49]. The EP and EPclin scores were also validated in node-positive, anthracycline +/− taxane chemotherapy-treated, HR+/HER2− patients in the GEICAM 9906 trial and appeared to be an independent prognostic factor for distant metastasis [50]. Even with nodal involvement, patients in the low-risk EPclin-based group had an excellent prognosis and might not benefit from extended adjuvant therapy. Prospective validation of such a strategy is currently being investigated in the EndoPredict © Extended Endocrine Trial (NCT04016935).

The Beast Cancer Index (BCI ©) assay combines two independently developed gene expression biomarkers: the molecular grade index which reflects both tumor grade and cell proliferation, and the H/I ratio which is an independent prognostic biomarker reflecting tumor proliferation and estrogen signaling [51]. The BCI © prognostic ability to predict both early and late distant recurrence was shown in the TransATAC cohort of post-menopausal node-negative breast cancer patients [52]. Another validation cohort included both pre- and post-menopausal patients, but the sample was too small to validate the BCI © assay for pre-menopausal women [53].

“Prospective-retrospective” long-term analyzes of breast cancer patients receiving hormone therapy for 5 years provide accurate estimations of late recurrence risk. However, a high risk of late recurrence is not necessarily correlated with a benefit for extended hormone therapy.

### 5.2. Prediction for Benefit of Extended Hormone Therapy

Sgroi and colleagues suggested that BCI © assay could predict benefit for extended hormone therapy: in the NCIC CTG MA.17 randomized clinical trial, patients categorized as BCI-high had a 67% reduction in risk of late recurrence with extended anti-aromatase treatment, while patients with BCI-low risk did not benefited from this extended hormonal adjuvant treatment [54]. A similar benefit of 65% reduction in recurrence risk with extended hormone therapy was shown for the BCI-high patients in a retrospective analysis of the prospective aTTom trial, contrary to BCI-low patients who did not benefit despite having positive nodes [55]. However, in a recent translational cohort from the NSABP B-42 trial, the BCI © H/I ratio taken apart failed to predict the benefit of extended anti-aromatase therapy in postmenopausal HR+ breast cancer [56] whereas the MammaPrint © assay predicted a significant benefit for low genomic-risk patients [57]. The first prospective data in the real life setting were provided by Sanft and colleagues who evaluated how the BCI © assay could impact the decision to extend adjuvant hormone therapy beyond 5 years: the BCI © results led to a change in physician treatment recommendation in 30% of patients (mainly against extended treatment), decreased the patients’ anxiety and decision conflict feelings and appeared to be cost-saving [58].

Overall, the BCI © assay seems to be the most promising signature to tailor indications for extended hormone therapy after breast cancer. Yet, more data are needed to increase the level of evidence, particularly in premenopausal women. Long-term prospective observational studies such as the RESCUE program (NCT03503799) will increase our knowledge. Prospective inclusion of gene expression signatures in future randomized clinical trials evaluating extended hormone therapy as adjuvant treatment for early breast cancer is warranted to formalize the predictive role of such assays.

## 6. Can We Tailor Radiation Therapy Decisions?

Radiation therapy after breast conserving surgery is known to improve locoregional and distant control [59]. However, this treatment is associated with both acute and long-term toxicities including cardiotoxicity, lung injury and second malignancies [60]. The question of omitting adjuvant radiation therapy in favorable-risk early breast cancer remains controversial, except in elderly women for whom this attitude is supported by several clinical trials and a meta-analysis [61]. The use of gene expression signatures could help to better discern which patients might benefit most from radiation therapy and which can be spared.

### 6.1. From Historical Biomarkers to Gene Expression Signatures

The approximation of breast cancer intrinsic subtypes, by immunohistochemical evaluation of hormonal receptors and HER2 expression, enabled a first classification of early breast cancer radiosensitivity. Extended follow-up of patients who received breast-conserving surgery and whole-breast radiation therapy revealed that local recurrence rates varied among molecular subtypes, from 0.8% and 1.5% in luminal A and B tumors, respectively, to 7.1% in basal and 8.4% in HER2− overexpressing tumors (at a time were trastuzumab was not used) [62]. However, a subgroup analysis of the DBCG82 trials evaluating post-mastectomy radiation demonstrated an overall benefit for HR+/HER2− luminal-like tumors only [63].

Better than surrogate immunohistochemistry-based classifications, the established gene expression signatures could play a role in the decision of adjuvant radiation therapy. The OncotypeDX © recurrence score was shown to be associated with a higher risk of locoregional recurrence among postmenopausal node-positive patients, regardless of the systemic adjuvant treatment [64]. The Prosigna ©/PAM50 assay was correlated to the locoregional recurrence risk in postmenopausal HR+/HER2− breast cancer but failed to predict the benefit of radiation therapy [65].

### 6.2. Development of Dedicated Radiosensitivity Signatures

The development of gene expression signatures able to predict tumor radiosensitivity is warranted to individualize the radiation therapy doses and indications [66]. Speers et al. developed a breast cancer specific Radiation Sensitivity Signature (RSS) [67]. This 51-genes signature, focused on cell-cycle control and DNA-damage response, was not correlated to the intrinsic breast cancer subtypes, but outperformed all clinically used clinicopathologic criteria to predict locoregional recurrence after breast-conserving surgery followed by radiation therapy. A prospective-retrospective analysis from randomized clinical trials evaluating the benefit of radiation therapy after breast conservative surgery is being conducted to corroborate its predictive ability [68].

More recently, Sjöström and colleagues developed the Adjuvant Radiotherapy Intensification Classifier (ARTIC) transcriptomic signature from the SwBCG91-RT trial, in which patients with node-negative stage I–II breast cancer were randomly assigned after breast-conservative surgery to receive whole-breast adjuvant radiation therapy or not [69]. The ARTIC signature was highly prognostic for locoregional recurrence in patients treated with radiation therapy and predicted radiation therapy benefit: whole-breast irradiation was effective in low-risk ARTIC scores with a 67% improvement of 10-year locoregional recurrence cumulative incidence but appeared insufficient in high-risk ARTIC scores, who might benefit from intensified treatment strategies [70].

Another approach has been to integrate the pan-cancer RSI Radiosensitivity signature to breast cancer subtypes. This was retrospectively addressed in patients treated with breast-conservating surgery followed by whole-breast radiation therapy with or without tumor-bed boost [71]: combination of RSI and molecular subtype was prognostic for local recurrence and identified a radioresistant subpopulation among triple-negative tumors who might benefit from radiation dose escalation.

### 6.3. Clinical Validation

Current evidence is insufficient to recommend omitting adjuvant radiation therapy only on the basis of gene expression signature results.

Several clinical trials are ongoing to integrate genomic-based prognostic and predictive signatures into locoregional radiation therapy decisions for early breast cancer patients. At least three prospective non-randomized studies will follow HR+/HER2− postmenopausal patients considered at low risk of recurrence after breast-conservative surgery for whom adjuvant radiation therapy will be avoided: PRECISION enrolls node-negative, T1, grade 1 or 2, Prosigna © low-ROR score tumors (NCT02653755); IDEA (Individualized Decisions for Endocrine Therapy Alone) selects patients with an OncotypeDX © Recurrence Score less than or equal to 18 (NCT02400190); and PRIMETIME aims to include 1500 patients with a “very low” IHC4+C score (measuring the protein levels of ER, PR, HER2 and Ki67 rather than gene expression) indicating a less than 5% risk of distant relapse at 10 years [72].

Non-inferiority randomized phase III clinical trials are needed for an optimal level of evidence. The EXPERT trial will use the Prosigna ©/PAM50 assay to select luminal A, stage I breast cancers and evaluate radiation therapy versus observation following breast-conservative surgery (NCT02889874). The MA39 TAILOR RT will focus on regional radiation therapy and will assign HR+/HER2- low-risk (defined by an OncotypeDX © Recurrence Score < 18) breast cancer patients with up to three positive axillary nodes to regional radiation therapy or observation (NCT03488693) [73].

## 7. Can Gene Expression Signatures Be Useful in the Neoadjuvant Setting?

Neoadjuvant chemotherapy has become a standard treatment in HER2-positive and triple-negative early breast cancer, with high rates of pathological complete response (pCR) and survival benefits [74]. In HR-positive/HER2-negative breast cancer, the benefit of neoadjuvant chemotherapy is unclear, whereas neoadjuvant hormone therapy might be a reasonable option with similar response rates and less toxicity [75]. Besides classical clinicopathological parameters, gene expression signatures appeared as an opportunity to tailor systemic neoadjuvant treatments.

### 7.1. HR+/HER2− Tumors

In retrospective studies, the OncotypeDX © high recurrence score results were associated with higher rates of pathological complete responses (pCR) to neoadjuvant chemotherapy in HR+/HER2− breast cancer, whereas low recurrence score results were associated with poorer responses [76,77]. A recently published prospective non-randomized study evaluated the impact of OncotypeDX © testing in HR+/HER2− patients previously selected by a multidisciplinary tumor board to receive neoadjuvant chemotherapy based on classical clinicopathological parameters: 64.7% of them had a low or intermediate risk recurrence score which enabled withdrawing the pre-testing indication of chemotherapy in 42% of patients; among the low or intermediate risk patients who even so received neoadjuvant chemotherapy, none had a major pathological response [78].

In parallel, low recurrence score results were shown to predict response to neoadjuvant hormone therapy in postmenopausal women [79,80,81] and to improve breast conservative surgery rates [80,81].

Taken together, the aforementioned studies support that OncotypeDX © recurrence score could guide the choice of neoadjuvant systemic therapy for HR+/HER2− breast cancers. Bear et al. conducted a pilot study in which low-risk patients received hormone therapy, high-risk patients received chemotherapy, and intermediate risk-patients were randomized to one or the other: this tailored strategy was shown to be feasible and led to similar breast conservative surgery rates in neoadjuvant hormone therapy-treated patients, whether they were of low or intermediate risks [82].

Other available gene expression signatures were evaluated in the neoadjuvant setting. Being strongly associated with hormone therapy sensitivity, the EndoPredict ©, MammaPrint © and BCI © assays were good candidates. A gene expression-based meta-analysis showed that whatever signature was used, most of the prediction of response to neoadjuvant chemotherapy was associated with genes linked to proliferation [83].

In HR+/HER2− cancers, the EndoPredict © score was predictive for systemic neoadjuvant treatment efficacy: EP high-risk tumors were more likely to respond to chemotherapy whereas EP low-risk tumors were more likely to benefit from neoadjuvant hormone therapy [84,85]. Head-to-head comparison showed that the 12-gene EndoPredict © score outperformed the 21-gene OncotypeDX © recurrence score to predict neoadjuvant chemotherapy efficacy in HR+/HER2− breast cancer [86]. In non-pCR tumors after neoadjuvant chemotherapy, persistent high-risk mEPclin score (combination of EP score and ypTN) predicted a higher risk of distance recurrence and mortality [87].

The MammaPrint © index [88,89], Prosigna ©/PAM50 assay [90] and Breast Cancer Index © [91,92] were also validated as significant predictors of response to neoadjuvant chemotherapy in predominantly HR+/HER2− populations. However, we still lack prospective data about their clinical use. The PLATO study (NCT03900637) is an ongoing phase 2 prospective study, using the MammaPrint © assay to guide neoadjuvant systemic treatment in stage I–IIIA, HR+/HER2− breast cancer patients inaccessible to frontline breast conservative surgery: the primary objective is a 15% increase of conversion rate to breast conservative surgery eligibility [93].

To summarize, all established signatures showed promising results to predict response to neoadjuvant systemic treatments, with strongest evidence for OncotypeDX © and EndoPredict © assays. The results from prospective studies such as PLATO trial are awaited with interest.

### 7.2. The Rise of Post-Neoadjvuant Biomarkers

In HR+/HER2− patients receiving hormone therapy in the neoadjuvant setting, post-treatment evolution of the Ki67 proliferation marker, alone or included in the PEPI score, was correlated with long term outcomes [94,95]. The combination of pre-treatment and post-treatment OncotypeDX © recurrence scores was also highly predictive of disease-free survival, and could differentiate patients at risk of early recurrence or mid/late recurrence [96]. Similarly, an ongoing study is monitoring changes in MammaPrint © risk under neoadjuvant hormone therapy (NCT04129216), and the DxCARTES trial is evaluating the ability of letrozole and palbociclib to decrease OncotypeDX © recurrence score or to perform pCR in pre-treatment intermediate and high recurrence scores patients (NCT03819010) [97].

### 7.3. Extension toward Triple-Negative Tumors

Beyond luminal tumors, gene expression signatures were further developed in more aggressive breast cancers. The Prosigna ©/PAM50 score was recently studied in early triple-negative breast cancer and may help to select patient for deescalated chemotherapy [98]. In the post-neoadjuvant setting, the ECOG-ACRIN EA1131 study aims at comparing capecitabine versus platinum-based chemotherapy in patients with residual disease and will evaluate the predictive impact of the PAM50 assay in such situation (NCT02445391).

However, the translation of established gene expression signatures—which were essentially developed in HR+/HER2− breast cancer populations—might not be optimal. Many processes are involved in chemosensitivity and chemoresistance, and they differentially account in each tumor subtype [83]. Zhao et al. developed a response probability score (RPS), which differentially involved markers of tumor cell proliferation rate, immune cell infiltration and stromal cell abundance in all-coming and triple-negative tumors [99].

Most established gene expression signatures were based upon expression levels of selected genes of interest. Modern biology techniques enable going further. The ongoing Breast-sign study (NCT03314870) will use a novel RNAseq technique to investigate levels of expression of several mRNA and miRNA linked to epithelial-mesenchymal transition and immune status, both in the tumor and in the plasma. As these two parameters are known to participate to chemoresistance in breast cancer [100], a specific signature might by helpful to avoid harmful neoadjuvant chemotherapies.

Current evidence is insufficient to advise using gene expression signatures in triple-negative tumors. Dedicated assays might be of high value in the future.

## 8. Which Role in the CDK4/6 Inhibitors Area?

CDK4/6 inhibitors have shown significant antitumor activity in advanced HR+/HER2− breast cancer [101]. Their benefit in early breast cancer remains controversial. In monarchE trial, adjunction of abemaciclib to adjuvant hormone therapy in HR+/HER2− high-risk early breast cancer significantly improved invasive disease-free survival, particularly in the premenopausal subset [102]. By contrast, the PALLAS trial failed to improve invasive disease-free survival with adjunction of palbociclib to adjuvant hormone therapy in stage II–III HR+/HER2− breast cancer [103]. In monarchE trial, high-risk patients were selected according to cTNM, tumor grade and centrally assessed Ki-67 [102]. In subgroup analyses, patients with Ki-67 ≥ 20% had a numerically better hazard ratio for efficacy outcome [104]. Specific predictive biomarkers are needed to optimize the selection of patients who might benefit most from CDK4/6 inhibitors in adjuvant or neoadjuvant setting.

### 8.1. Predictive Ability

A translational analysis of the MONALEESA phase III studies recently offered a first insight in molecular prediction of CDK4/6 inhibitors [105]. In this study, Prat and colleagues evaluated the correlation between molecular intrinsic subtypes (determined with a custom gene panel including 36 of the 50 Prosigna © assay genes) and clinical response to ribociclib in advanced breast cancer. All subtypes except the basal-like (accounting for 2.6% of this HR+/HER2− population) derived from significant progression-free survival improvement. Of note, the HER2-enriched subtype (accounting for 12.7%) had the worst prognosis but benefitted the most from ribociclib with a 61% relative improvement of PFS.

In early breast cancer, data were lacking about the predictive role of gene expression signatures towards CDK4/6 inhibitors efficacy. The NeoPAL was a randomized phase II study evaluating letrozole and palbociclib versus chemotherapy as neoadjuvant treatment for high-risk luminal breast cancer. This study successfully used the Prosigna ©/PAM50 signature to select patients (only Prosigna-defined luminal B, or luminal A and node-positive, tumors could be included) but failed to demonstrate any improvement of pathological response nor a correlation between risk of recurrence score (ROR) and efficacy outcome [106].

Current evidence is insufficient to draw any conclusion about the ability for gene expression signatures to identify patients who might benefit from CDK4/6 inhibitors in the neoadjuvant or adjuvant setting. Results of ongoing prospective trials are awaited. For instance, the NSABP FB-13 study (NCT03628066) is stratifying patients according to their tumor OncotypeDX © recurrence score to evaluate the efficacy of neoadjuvant palbociclib with letrozole and ovarian suppression in premenopausal HR+/HER2− breast cancer [107]. The POETIC-A trial (NCT04584853) is based on a different approach and selects patients whose tumor Ki67 level does not drop after 2 weeks of anti-aromatase neoadjuvant treatment and explores the benefit of adding abemaciclib as post-neoadjuvant treatment. Interestingly, a translational analysis will allow prospectively evaluating a dedicated predictive signature called Aromatase Inhibitor Resistant-CDK4/6 Inhibitor Sensitive (AIR-CIS).

### 8.2. Molecular Downstaging

Besides their potential utility to select high-risk or highly sensitive patients who might benefit most from CDK4/6 inhibitors in early breast cancer, gene expression signatures have recently been intended for measuring treatment activity as a new endpoint. Indeed, the conversion from an aggressive” (i.e., luminal B) subtype to a more indolent (i.e., luminal A) subtype could represent a benefic transformation of the tumor intrinsic natural evolution. This “molecular downstaging” theory was first approached by the decrease of Ki67 under hormone therapy [94]. The CORALLEEN phase II trial showed that combination of ribociclib and letrozole could be as effective as neoadjuvant standard chemotherapy to convert PAM50 high-ROR tumors to low-ROR in postmenopausal luminal early breast cancer [108].

Molecular downstaging appears promising but clinical benefit needs to be demonstrated in further studies [109]. The NCT03969121 phase III study is studying both clinical and molecular responses to palbociclib and hormone therapy as neoadjuvant treatment of HR+/HER2− breast cancer and will bring interesting data.

## 9. How Do Gene Expression Signatures Influence Clinical Decisions?

### 9.1. Decreasing Chemotherapy Indications

The primary goal of gene expression signatures is to spare a potentially toxic treatment to patients for whom it is not likely to be beneficial [110]. In the MINDACT trial, the Prosigna ©/PAM50 assay enabled a 46% decrease of chemotherapy indications in a clinical-high risk HR+/HER2− population [6]. However, real life data were needed to corroborate this clinical utility.

A pooled analysis of four prospective European studies assessing the impact of using the OncotypeDX © recurrence score in node-negative HR+/HER2− early breast cancer demonstrated that chemotherapy recommendation rate decreased from 55% to 34% [111,112]. Similar results were reported in node-negative [113] but also node-positive early breast cancer [114,115,116]. The Prosigna © PAM50 [117,118,119,120] and EndoPredict © [121] assays also demonstrated a 20–35% decreasing of chemotherapy indications in HR+/HER2− early breast cancer.

### 9.2. Improving the Decision-Making Process

Several of the above-mentioned decision-making studies evaluated as secondary objectives the psychological impact of gene expression signatures. They demonstrated an improvement of physicians’ confidence regarding treatment recommendations [111,113,119,120], as well as a decreasing in patients anxiety and decisional conflict [112,116,117,118,120,121]. However, at least two studies showed that an unfavorable genomic result might increase anxiety, especially in a two-step decision making process [118,121]. Concerns about their cost, inappropriate use and over-reliance are also rising [122].

It is established that cancer patients who assume an active role in treatment decision making have a better quality of life [123]. Gene expression signatures might be a useful tool in the decision process to better inform and involve the patient. The patients’ interest is high: a large majority of women previously treated for early breast cancer would “definitely” want to be tested when being presented to hypothetical scenarios implementing gene expression signatures [124].

Yet, several studies highlighted the risk of over-reliance: some patients might believe that the genomic test is more important than clinical parameters and might rely only on it [125,126]. On the contrary, women who are confident about their treatment decisions might be unsettled in case of discordant result [127].

The patient’s comprehension is another issue. Breast cancer patients who benefitted from such assays might wrongly interpret the results due to semantic confusions and lack of information [128,129]. The rate of patients able to describe or recall their recurrence risk based on a gene expression signature ranges between 33% and 71%, and might depend on how it was reported and explained [130,131]. Several reporting methods were developed and compared: a newly developed risk-continuum format decreased the misinterpretation rate from 17% to 5% [132], and a combined oral and printed information was shown to improve patients’ knowledge and comprehension [129].

The MEND2 trial (NCT03183050) is currently evaluating the interest of a dedicated tool to improve communication and decision making in the context of patients receiving OncotypeDX © testing. Future biomarker studies will have to prospectively assess patient’s comprehension, involvement in treatment decisions, long-term adherence and quality of life.

## 10. Are Gene Expression Signatures Cost-Effective?

The development of gene expression signatures raised the question of their cost-effectiveness. Rouzier and colleagues published in 2013 a systematic review of health economic analyzes from 29 publications evaluating the Oncotype DX © and the MammaPrint © assays [133]. Both signatures were globally cost-effective and sometimes cost-saving. The cost-benefit ratio was driven by the reduction in chemotherapy prescription, and therefore greater in the US setting where chemotherapy was more frequently recommended and more expensive.

Other studies suggested that strategies based on gene expression signatures were associated with a higher cost [134,135], especially in an era when taxane-based regimens became cheaper, whereas the cost of genomic assays remained high. However the prospective PREGECAM medico-economic multicenter study recently retrieved favorable outcomes in terms of quality-adjusted life-years and cost-efficacy [136]. Well-designed prospective cost-efficacy evaluations are still needed to address this controversial debate.

## 11. Conclusions

Gene expression signatures were initially designed to take into account tumor biology for adjuvant chemotherapy decision in early breast cancer. Recent phase III randomized clinical trials provided high level evidence to support their use: the MINDACT trial confirmed that patients with clinical-high risk but Mammaprint © genomic-low risk could be spared from adjuvant chemotherapy regardless of their hormone-receptor expression, HER2-status and nodal involvement; the TAILORx trial supported omission of adjuvant chemotherapy for HR+/HER2− node-negative patients with OncotypeDX © recurrence score ≤ 25 (with a potential concern for intermediate-high risks and premenopausal patients); and the WSG-planB trial showed that adjuvant chemotherapy could also be omitted for patients with up to three involved nodes when recurrence score is ≤11. However, despite the randomized design, these studies had some limitations. TAILORx, WSG-plan B and ongoing RxPonder trials assumed that Oncotype DX © was the reference method, and did not compare it with validated prognostic models. Only the MINDACT trial aimed at comparing MammaPrint © assay with the Adjuvant! Online model, which limited conclusions to the discordant clinical-high risk/MammaPrint © low risk subgroup. Prospective randomized trials are still needed to offer clear and strong evidence. The OPTIMA de-escalation trial will evaluate the ability of the Prosigna ©/PAM50 assay to tailor adjuvant therapy for HR+/HER2− high-clinical risk breast cancer (ISRCTN42400492). However, despite an innovative adaptive design and digital communication efforts, the trial is experiencing recruitment issues reflecting the complexity of modifying practice [137].

Retrospective studies provided a large amount of data about the use of gene expression assays in several situations which were out of the scope of pivotal clinical trials. Physicians might feel unconfident in their daily practice. Our review suggests that commercially available gene expression signatures remain relevant in specific subgroups such as elderly and non-Caucasian patients. However, caution must be exercised for premenopausal women, considering subgroup analyzes of TAILORx and RxPONDER trials.

Apart from chemotherapy indications, gene expression signatures are increasingly used to tailor other adjuvant treatments decisions for early breast cancer.

Regarding extended hormone therapy, BCI © assay seems to be the most promising signature, but prospective data are awaited to validate its use in daily practice.

Current evidence does not support for the use of one or another established signatures to individualize adjuvant radiation therapy indications. Results from several non-inferiority randomized phase III trials are awaited with great interest.

In the neoadjuvant setting, all established signatures showed promising results to predict response to systemic treatments, with strongest evidence for OncotypeDX © and EndoPredict © assays. Prospective analyzes are needed to support their use in daily practice.

In the future, gene expression signatures could be used to identify patients who might benefit from CDK4/6 inhibitors as adjuvant treatment. “Molecular downstaging” might be an interesting early biomarker for CDK4/6 inhibitors sensitivity in the neoadjuvant setting.

Besides demonstration of clinical utility in decision making process and optimization of survival outcomes, efforts in evaluating quality-of-life benefit and cost-effectiveness ought to be pursued. The results of these assays are often multilayered rather than binary. Their optimum use and communication of their outcomes to patients require greater understanding by the medical community, including acknowledgment of areas of uncertainty.

## Figures and Tables

**Table 1:** Main characteristics of the five commercially available gene expression signatures.

Signature	Genes	Clinical Studies	Applicability
OncotypeDX ©	21 (16 cancer related + 5 reference genes)	NSABP B14, NSABP B20 NSABP B28, SWOG 8814 TransATAC, ECOG E2197 TAILORx * RxPONDER * WSG-planB *	HR+/HER2− N−/+ Pre/post-menopausal
MammaPrint ©	70 genes	TRANSBIG RASTER * MINDACT * I-SPY2 *	HR+/HER2−/+ N−/+ Pre/post-menopausal
Prosigna ©	58 (50 cancer related + 8 reference genes)	ABCSG-8 TransATAC	HR+/HER2− N−/+ Post-menopausal
EndoPredict ©	12 (8 cancer related + 4 reference genes)	GEICAM 9906 ABCSG-6 ABCSG-8	HR+/HER2− N−/+ Post-menopausal
Breast Cancer Index ©	7 genes	Stockolm trial TransATAC NSABP 42 MA. 17	HR+/HER2− N−/+ Post-menopausal

* Prospective trial.

**Table 2:** Main characteristics of recent phase III randomized clinical trials.

	MINDACT (*n* = 6693)	TAILORx (*n* = 9719)	WSG-Plan B (*n* = 2642)
Study characteristics			
**Assay**	MammaPrint ©	OncotypeDX ©	OncotypeDX ©
**Eligible patients**	invasive BC, T1-T3, 0–3N+	invasive HR+/HER2− BC, N0, adjuvant CT indication according to NCCN guidelines	invasive HER2− BC, N+ or high risk (≥T2, grade 2–3, age < 35)
**Groups and randomization**	4 groups according to Clinical risk (Adjuvant! Online) and Genomic risk (MammaPrint ©): ▪ C-low/G-low (41%): no CT ▪ C-low/G-high (8.8%): randomized (CT vs. not CT) ▪ C-high/G-low (23.2%): randomized (CT vs. not CT) ▪ C-high/G-high (27%): CT	3 groups according to OncotypeDX ©: ▪ RS ≤ 10 (17%): no CT ▪ RS 11–25 (69%): randomized (CT vs. no CT) ▪ RS ≥ 26 (14%): CT	3 groups according to OncotypeDX ©: ▪ RS ≤ 11 (17.8%): randomized (CT vs. no CT) ▪ RS 12–25 (61.4%): CT ▪ RS ≥ 26 (20.8%): CT
**Primary endpoint**	5-year DFMS ≥ 92% for C-high/G-low pts who did not receive CT	IDFS non-inferiority of HT alone vs. CT + HT in mid-risk patients	IDFS of low-risk patients treated with HT alone
**Population main characteristics**			
**Age**	32.2% < 50 years	31.4% < 50 years	median 56 years
**Grade 3**	28.8%	17.2%	31.2%
**HER2+/TNBC**	9.5%/9.6%	0%/0%	0%/2.4%
**N+**	21%	0%	35.2% pN1/6% pN2–3

BC, breast cancer; CT, chemotherapy; DFMS, distant metastasis-free survival; HT, hormone therapy; IDFS, invasive disease-free survival; NCCN, national comprehensive cancer network; TNBC, triple-negative breast cancer.

**Table 3:** Main characteristics of ongoing clinical trials.

Study (ID) *Assay*	Design/Inclusion Criteria	Target/Primary Endpoint	Method
**Specific populations**			
**GERICO 11/ASTER 70s** (NCT01564056) *Genomic Grade test*	randomized phase III trial	2000	Enrollment after breast surgery and test with Genomic Grade test. High-risk patients are randomized between HT alone or chemotherapy followed by HT, whereas low-risk or contra-indicated patients are followed in an observational cohort receiving HT alone.
	invasive HR+/HER2− BC, N0 or N+, age ≥ 70, PS ≤ 2	OS	
**Extended hormone therapy**			
**EXET** (NCT04016935) *EndoPredict ©*	prospective cohort study	2800	Enrollment near the 5-year post-diagnosis time point and test with EndoPredict ©. Choice of extended HT at the discretion of the patient and his physician. Follow-up for 6 years.
	invasive HR+/HER2− BC, stage I–III, 0–3 N+, possible adjuvant CT but no neoadjuvant CT	DRFS	
**RESCUE** (NCT03503799) *EndoPredict ©*	prospective cohort study	1200	Enrollment within 6 months after breast surgery. No impact on clinical decision. Follow-up for 10 years.
	invasive HR+/HER2− BC, stage I–III, 0–3 N+	DRFS	
**Radiation therapy**			
**Radiotype DX** (IRAS162496) *Radiotype DX ©*	retrospective analysis of prospectively collected data	840	Anonymised tissues from patients enrolled in the Scottish Conservation Trial and PRIME I Trial tested with Radiotype DX © and matched with long term clinical outcomes.
	invasive HR+/HER2− BC, T0–T2, N0, age ≥ 65	IBTR	
**PRECISION** (NCT02653755) *Prosigna ©*	non-randomized comparative phase II trial	672	Enrollment after breast conservative surgery and test with Prosigna ©. Patients are eligible to omit adjuvant radiotherapy only if genomic score is low. Follow-up for 5 years.
	invasive HR+/HER2− BC, grade 1–2, T1, N0, age 50–75, PS ≤ 2	IBTR	
**IDEA** (NCT02400190) *OncotypeDX ©*	prospective cohort study	202	Enrollment after breast conservative surgery and test with OncotypeDX ©. Eligible to omit adjuvant radiotherapy only if the genomic score is ≤18. Follow-up for 5 years.
	invasive HR+/HER2− BC, T1, N0, postmenopausal status, OncotypeDX © recurrence score ≤ 18	IBTR	
**PRIMETIME** (IRAS190307) *IHC4+C score*	prospective cohort study	2400	Screening before breast conservative surgery, definitive enrollment after surgery and test with IHC4+C score. Recommended to omit adjuvant radiotherapy only if ‘very low’ risk. Follow-up for 10 years.
	invasive HR+/HER2− BC, grade 1–2, T1, N0, age ≥ 60	IBTR	
**EXPERT** (NCT02889874) *Prosigna ©*	non-inferiority randomized phase III trial	1167	Enrollment after breast surgery and test with Prosigna ©. High-risk (ROR > 60) patients are excluded; low-risk (ROR score ≤ 60) patients are randomized between standard of care (HT and radiotherapy) or avoidance of radiotherapy (HT only).
	invasive HR+/HER2− BC, grade 1–2, T1, N0, age ≥ 50, PS ≤ 2, Prosigna © ROR score ≤ 60	IBTR	
**MA39 TAILOR RT** (NCT03488693) *OncotypeDX ©*	non-inferiority randomized phase III trial	2140	Enrollment after breast surgery and test with OncotypeDX ©. High-risk (RS > 25) patients are excluded, low-risk (RS ≤ 25) patients are randomized between standard of care (HT and radiotherapy including regional nodes) or avoidance of regional radiotherapy (HT, breast or chest wall irradiation if indicated, no lymph node irradiation).
	invasive HR+/HER2− BC, T1–T2 and ≤ 3 N+ or T3N0, age ≥ 35, PS ≤ 2, OncotypeDX © ROR score ≤ 25	BCRFI	
**Neoadjuvant treatments**			
**PLATO** (NCT03900637) *MammaPrint ©*	non-randomized comparative phase II trial	122	Enrollment in the neoadjuvant setting and test with MammaPrint ©. High-risk patients are treated with neoadjuvant chemotherapy, whereas low-risk patients are treated with neoadjuvant HT. Follow-up for 5 years.
	invasive HR+/HER2− BC, stage I–IIIA, ineligible for breast conservative surgery, age ≥ 19, PS ≤ 2	conversion rate	
**DxCARTES** (NCT03819010) *OncotypeDX ©*	non-comparative phase II trial	66	Enrollment in the neoadjuvant setting and test with OncotypeDX ©. All patients are treated with palbociclib + HT for 6 cycles before breast surgery with a second OncotypeDX © testing. Follow-up for 6 months.
	invasive HR+/HER2− BC, Ki67 ≥ 20, T2–T4, N0–N2, age ≥ 18, PS ≤ 1	Recurrence score	
**NSABP FB-13** (NCT03628066) *OncotypeDX ©*	non-comparative phase II trial	24	Enrollment in the neoadjuvant setting and test with OncotypeDX © to be stratified into one of two cohorts (recurrence score < 11 versus 11–25). All patients are treated with palbociclib + HT. Biopsy after 6 weeks of therapy: patients with a persistent Ki67 ≥ 10 will permanently discontinue and begin neoadjuvant chemotherapy or proceed to surgery (at the discretion of treating physician); patients with Ki67 < 10 will continue for a total of 6 cycles and a third Ki67 assessment will be performed at the time of surgery. Follow-up for 6 months.
	invasive HR+/HER2− BC, T2–T4, suitable for neoadjuvant HT, premenopausal status, PS ≤ 1, OncotypeDX © recurrence score < 26	Ki67	
**POETIC-A** (NCT04584853) *AIR-CIS*	randomized phase III trial	2500	Enrollment in the neoadjuvant setting and treatment with HT for 2 weeks before surgery. If Ki67 level does not drop at the time of surgery, patients are randomized between HT alone or abemaciclib (2 years) + HT as adjuvant treatment. Translational analysis will evaluate the Aromatase Inhibitor Resistant-CDK4/6 Inhibitor Sensitive (AIR-CIS) dedicated predictive signature.
	invasive HR+/HER2− BC, ≥1.5 cm, grade 3 and/or Ki67 ≥ 20%, postmenopausal status	BCRFI	
**Adjuvant chemotherapy**			
**OPTIMA** (ISRCTN 42400492) *Prosigna ©*	non-inferiority randomized phase III trial	4500	Enrollment after breast surgery and randomization between receiving standard treatment (chemotherapy followed by HT) or to undergo Prosigna © testing (those with high-score tumors will receive standard treatment whilst those with low-score tumors will be treated with HT alone). Follow-up for 10 years.
	invasive HR+/HER2− BC, pN1–2 or pN1mi with pT ≥ 20 mm or pN0 with pT ≥ 30 mm	IDFS	
**RxPONDER** (NCT01272037) *OncotypeDX ©*	randomized phase III trial	10 000	Enrollment after breast surgery and test with OncotypeDX ©. Only patients with recurrence score ≤ 25 are eligible and will be randomized between HT alone or HT + adjuvant chemotherapy. Follow-up for 15 years.
	invasive HR+/HER2− BC, 1–3 N+, recurrence score ≤ 25	IDFS	

BC, breast cancer; BCRFI, breast cancer recurrence-free interval; CT, chemotherapy; DRFS, distant recurrence-free survival; HT, hormone therapy; IBTR, ipsilateral breast tumor recurrence; IDFS, invasive disease-free survival; OS, overall survival.
